# Supplementary figures and images for: Subacute SARS-CoV-2 replication can be controlled in the absence of CD8+ T cells in cynomolgus macaques
Source: PLoS Pathog. 2021 Jul 19;17(7):e1009668. doi: 10.1371/journal.ppat.1009668 (PMC8321216; doi:10.1371/journal.ppat.1009668)

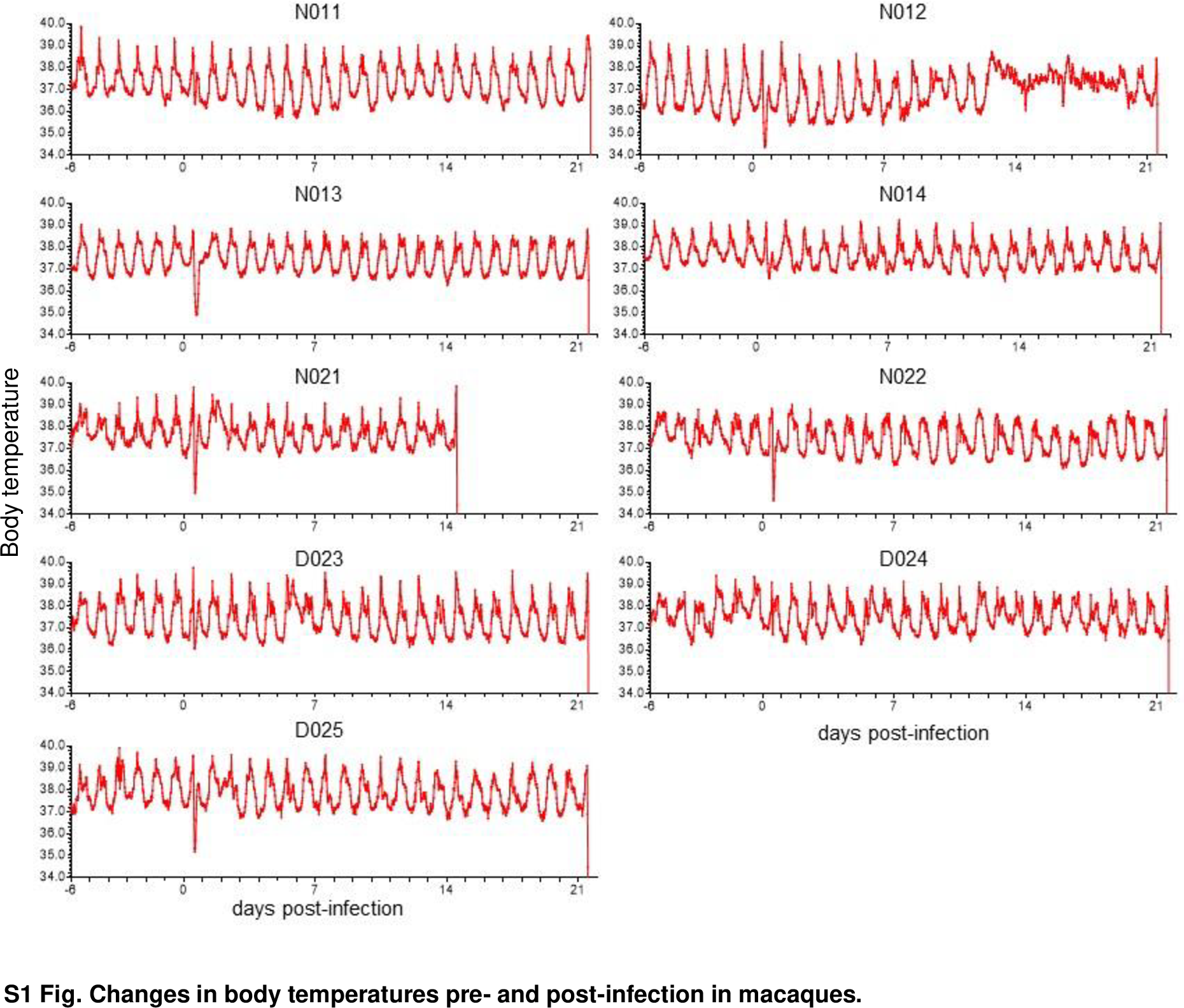

Supplement: S1 Fig — (TIF) [file ppat.1009668.s001.tif]

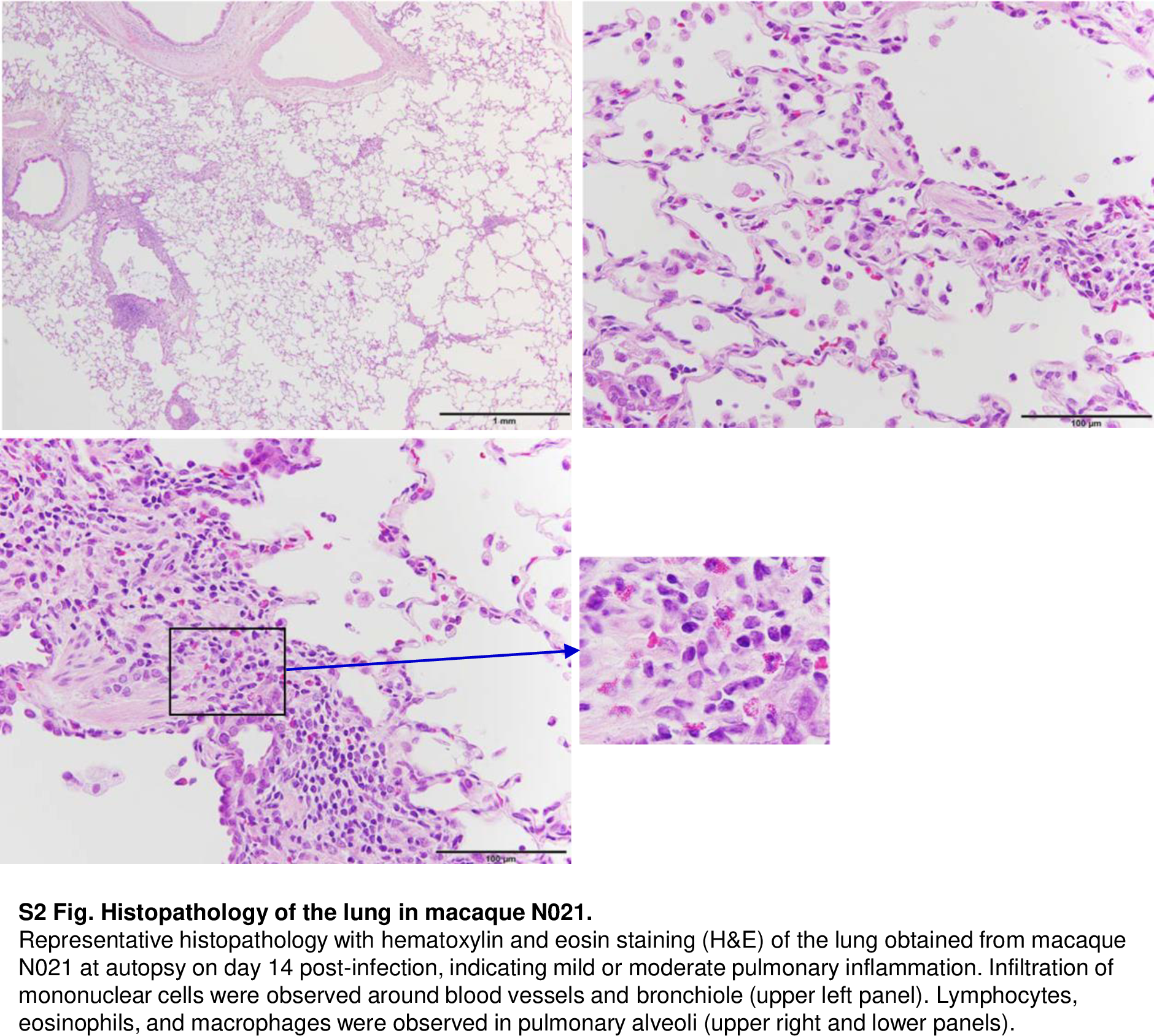

Supplement: S2 Fig — Representative histopathology with hematoxylin and eosin staining (H&E) of the lung obtained from macaque N021 at autopsy on day 14 post-infection, indicating mild or moderate pulmonary inflammation. Infiltration of mononuclear cells were observed around blood vessels and bronchiole (upper left panel). Lymphocytes, eosinophils, and macrophages were observed in pulmonary alveoli (upper right and lower panels). (TIF) [file ppat.1009668.s002.tif]

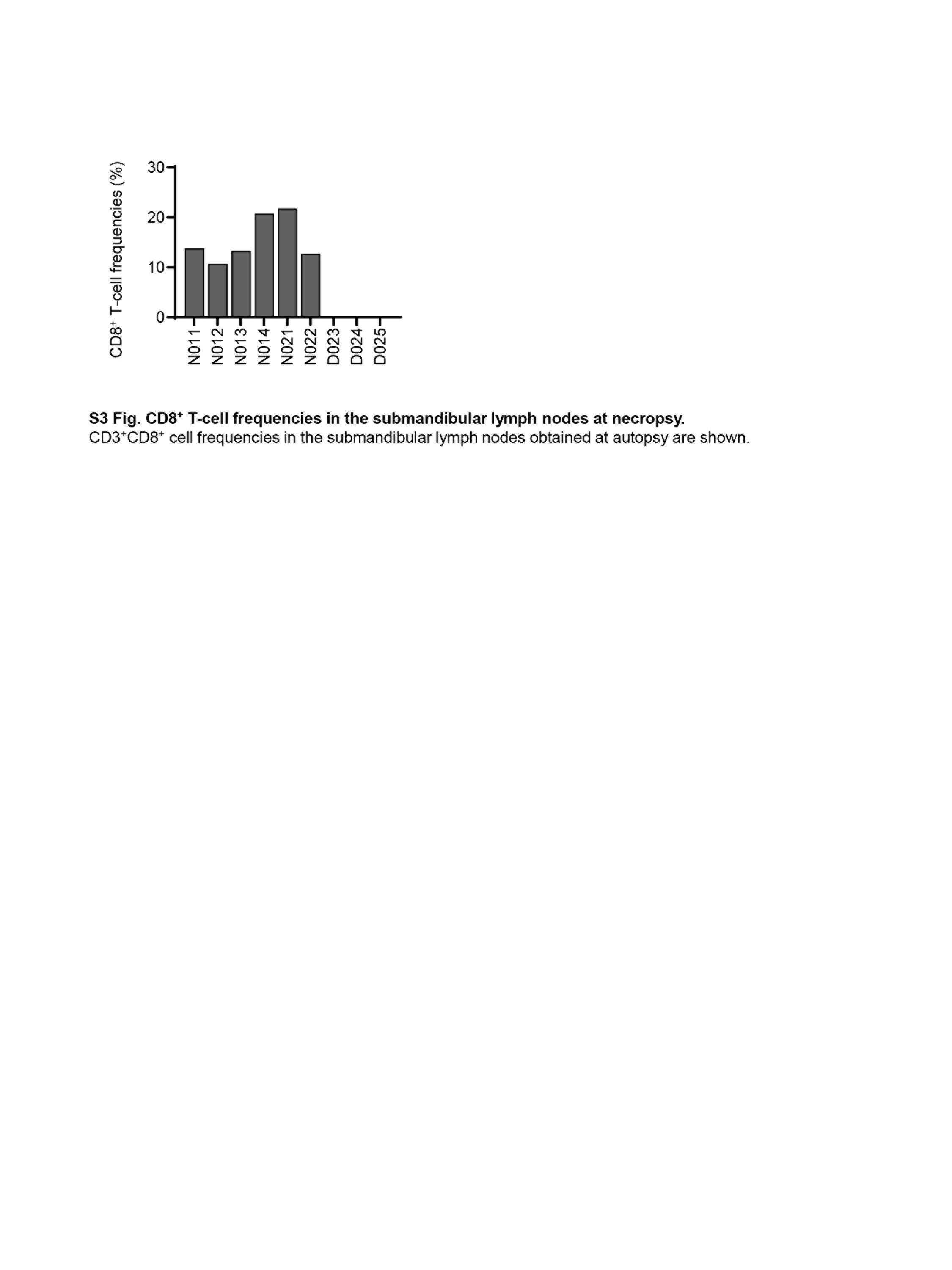

Supplement: S3 Fig — CD3+CD8+ cell frequencies in the submandibular lymph nodes obtained at autopsy are shown. (TIF) [file ppat.1009668.s003.tif]
