# Supplementary material for: Subacute SARS-CoV-2 replication can be controlled in the absence of CD8+ T cells in cynomolgus macaques
Source: PLoS Pathog. 2021 Jul 19;17(7):e1009668. doi: 10.1371/journal.ppat.1009668 (PMC8321216; doi:10.1371/journal.ppat.1009668)
Supplement: Attachment — Submitted filename: Nomura PP_Response to Comments.docx [file ppat.1009668.s004.docx]

**Responses to the Reviewers' comments (PPATHOGENS-D-21-01073)**

We have appreciated the Editors' and Reviewers' comments. The following are our responses to the comments. We hope that our explanations and revisions are satisfactory.

**Reviewer #1**

*(Summary)*

*Nomura et al CD8 deplete 3 macaques 5 days after SARS-CoV-2 infection and show the profile of resolution of infection is similar in these 3 macaques to 5 controls. The work is well done and of interest to the COVID field grappling with a role if any for CD8 T cells in control of COVID.*

*(Comments)*

- *Although acknowledged by the authors, the numbers of macaques and groups are very limited. I felt a more definitive data set would have perhaps considered a B cell depletion positive control.*

Response: We believe that B cell depletion is not required for our conclusion but may provide results regarding the role on B cell responses. We acknowledge this suggestion, which could be an issue to be addressed in the next study in future.

- *Only one time point of depletion was studied. I felt that day 5 was a reasonable stab at showing the importance of control, but it remains possible it was too late.*

Response: Our results on viral RNA and virus recovery from pharyngeal swabs indicate that viral replication was not contained at the time point of CD8 depletion. Furthermore, CD8^+^ T-cell responses in PBMCs were undetectable at week 1 but became detectable at week 2 in most animals. We thus believe that it is reasonable to reach our conclusion by CD8 depletion on day 5. We have appreciated this comment and have added a discussion related to this point (L.199 & L.206).

- *The role of CD8 T cells, if any, may be more important in prevention of severe disease, which macaques rarely suffer from. An alternate role may be in control of reinfection, as suggested by Barouch et al (albeit with limited data).*

Response: We agree to this point (which was briefly mentioned in Discussion). We have revised the Discussion (L.206) to mention this point more clearly.

**Reviewer #2**

*(Summary)*

*This study uses a macaque model with intranasal SARS-CoV-2 exposure to assess the role of CD8+ T-cells in viral control. The results demonstrate that CD8+ T cell depletion does not impact viral clearance during SARS-CoV-2 primary infection. The manuscript is well written, but could be improved by addressing some concerns related to study design and interpretation of the results.*

*1. The investigators should clarify the apparent timing issue where viral clearance occurs predominantly by 10 days post-infection, while induction of systemic CD8+ T cell responses occurs by 14 days post-infection. Their proposed temporal sequence of T-cell and B-cell activity in the primary infection should be provided in a diagram to illustrate the possible relationship with viral clearance.*

Response: As indicated by this comment, CD8^+^ T-cell responses mostly became detectable at week 2, suggesting that CD8^+^ T-cell responses may not play a central role in the control of peak viral load but contribute to the containment of viral replication after that in primary infection. However, it is difficult to mention how innate and/or B-cell responses can work against viral replication in the acute phase because this study did not focus on these responses. We have appreciated this comment and have revised the Discussion (L.206).

*2. The investigators should comment on whether there is actual depletion of CD8+ T cells in the tissues, especially at sites of SARS-CoV-2 replication. Looking at tissue specific CD8+T cells responses would be more informative considering where the virus replicates. This is demonstrated by authors own data on animal N013 that has no detectable systemic CD8+T cell responses but has responses as well as viral replication in submandibular lymph nodes.*

Responses: We have appreciated this comment and have added S3 Fig confirming CD8^+^ T-cell depletion in the submandibular lymph nodes obtained at necropsy (L.184).

*3. While the investigators find systemic SARS-CoV-2 specific CD8+ T cell responses in untreated macaques, the responses have a wide range. Even in the 5 animals examined, a wide range of responses can be noticed from no response (N013) to some response at d14. Having only 3 animals in the active arm and a wide range of responses in the control animals are two important limitations impacting each other in the interpretation of the results that the investigators should comment on.*

Response: We have appreciate this comment and have revised the Discussion (L.260).

*4. Since cellular immunity may play a more important role in clearing/preventing subsequent infections and controlling outcome in severe/uncontrolled infections, the investigators should comment on the need for further studies using a reinfection model to assess the role of CD8+ T cell responses in viral clearance.*

Response: We agree to this point (which was briefly mentioned in Discussion). We have revised the Discussion (L.206) to mention this point more clearly.

*5. Statements in lines 203-205 and 250-252 need to be adjusted to clearly state that the observations are only relevant for primary, controlled SARS-CoV-2 infections.*

Response: We have revised these sentences (L.216 & L.265) according to this comment.

**Reviewer #3**

*(Summary)*

*Nomura and colleagues assess the impact of CD8 T cell depletion on the control of subacute SARS-CoV-2 replication in a small cohort of cynomolgus macaques. They report that when CD8 depletion is performed 5-7 days after SARS-CoV-2 infection, there is no demonstrable impact on viral replication compared to control animals. They correctly surmise in the discussion that while this does not dispute a potential role for vaccine-induced CD8 T cells in protection from infection, the data suggest that CD8 T cells are not strictly required to control low-level viral replication in the pharyngeal mucosa.*

*The role for T cells in determining COVID-19 severity or vaccine-induced protection is a topic of significant interest, which can be challenging to conclusively assess in human cohorts. While the authors note the limitations of the current study, including the sample size, non-human primate studies are a valuable pre-clinical model to assess the contributions of various immune cells to viral control. The manuscript is well-written and presents the data and conclusions in a clear and concise manner. I have no substantial concerns about the study, but some additional discussion points would enhance its message (see below).*

*(Minor Issues)*

*One of the major considerations for the role of CD8 T cells in controlling viral replication during primary infection versus the contribution of T cells to protection from re-infection includes the kinetics of the T cell response. Based on the data presented in Figure 5, it is relatively clear that primary, subacute infection is unlikely to induce detectable CD8 responses prior to day 14 in most animals. Given how early viral replication peaks in this animal model, it seems likely that the primary CD8 response simply occurs too late to contribute to viral control. I think the discussion could more explicitly point this out, and perhaps report what is known about similar kinetics in humans.*

Response: As indicated by this comment, CD8^+^ T-cell responses may not play a central role in the control of peak viral load but contribute to the containment of viral replication after that in primary infection. We have appreciated this comment and have revised the Discussion (L.206).
